# Intraoperative delivery of the Notch ligand Jagged-1 regenerates appendicular and craniofacial bone defects

**DOI:** 10.1038/s41536-017-0037-9

**Published:** 2017-12-15

**Authors:** Daniel W. Youngstrom, Rafael Senos, Robert L. Zondervan, Jack D. Brodeur, Austin R. Lints, Devin R. Young, Troy L. Mitchell, Megan E. Moore, Marc H. Myers, Wei-Ju Tseng, Kathleen M. Loomes, Kurt D. Hankenson

**Affiliations:** 1 Orthopaedic Research Laboratories, Department of Orthopaedic Surgery, University of Michigan Medical School, Ann Arbor, MI USA; 2 Department of Physiology, Michigan State University College of Osteopathic Medicine, East Lansing, MI USA; 3 Department of Orthopaedic Surgery, University of Pennsylvania Perelman School of Medicine, Philadelphia, PA USA; 4 Division of Gastroenterology, Hepatology and Nutrition, The Children’s Hospital of Philadelphia, Philadelphia, PA USA

## Abstract

Each year, 33% of US citizens suffer from a musculoskeletal condition that requires medical intervention, with direct medical costs approaching $1 trillion USD per year. Despite the ubiquity of skeletal dysfunction, there are currently limited safe and efficacious bone growth factors in clinical use. Notch is a cell–cell communication pathway that regulates self-renewal and differentiation within the mesenchymal/osteoblast lineage. The principal Notch ligand in bone, Jagged-1, is a potent osteoinductive protein that positively regulates post-traumatic bone healing in animals. This report describes the temporal regulation of Notch during intramembranous bone formation using marrow ablation as a model system and demonstrates decreased bone formation following disruption of Jagged-1 in mesenchymal progenitor cells. Notch gain-of-function using recombinant Jagged-1 protein on collagen scaffolds promotes healing of craniofacial (calvarial) and appendicular (femoral) surgical defects in both mice and rats. Localized delivery of Jagged-1 promotes bone apposition and defect healing, while avoiding the diffuse bone hypertrophy characteristic of the clinically problematic bone morphogenetic proteins. It is concluded that Jagged-1 is a bone-anabolic agent with therapeutic potential for regenerating traumatic or congenital bone defects.

## Jagged-1 helps heal fractures faster

Localized and temporary delivery of the protein Jagged-1 promotes bone regeneration in rodents. Despite the large incidence of bone injuries in humans, current therapies to stimulate bone growth can have serious side effects. Kurt Hankenson at the University of Michigan, US, and colleagues have been investigating ways to stimulate Notch receptors, key regulators of bone formation during development. They found that levels of the Notch receptor’s binding partner Jagged-1 were significantly increased after bone injury and that disruption of Jagged-1 prevented bone healing in mice. Bone repair in both mice and rats was significantly improved following delivery of Jagged-1 on a collagen scaffold to the site of injury compared with controls. These findings could pave the way for safer and more efficient therapies to repair bone damage due to injury or inherited bone diseases.

## Introduction

Fractures, congenital defects, and malignancies of the cranial, axial, and appendicular skeleton present a common challenge; however, current therapies aimed at promoting bone regeneration have significant shortcomings. By the age of 50, 50% of women and 20% of men will have suffered a fracture.^1^ Up to 12% of tibia shaft fractures experience nonunion,^2^ and approximately 25% of bone allografts to repair the resection of malignant bone tumors fail.^3^ Indeed, the direct medical costs of osteoporosis-related fractures alone in the United States exceeds $20 billion annually.^4^ The total societal burden of these injuries, which includes healthcare utilization, reduced quality/quantity of life and opiate abuse, will continue to rise with ageing and the prevalence of comorbidities.^5^ The bone morphogenetic proteins (BMPs) were once championed as versatile skeletal growth factors and the answer to the bone healing problem, but the widespread off-label clinical use of BMPs contributed to an increasingly poor safety record and a pronounced drop in their utilization.^6^ Thus, there remains a major unmet public health need for alternative and/or complimentary bone-anabolic factors that can promote healing of high-risk fractures or volumetric bone defects.

Notch is an evolutionarily conserved cell-cell signaling pathway that, in mammals, has five ligands (Jagged-1, Jagged-2, Delta-like-1, Delta-like-3, Delta-like-4) and four receptors (Notch1–4). Upon ligand binding, Notch receptors activate by proteolytic cleavage of their intracellular domains (NICD), which translocate to the nucleus where they function as transcription factors for a variety of genes, including those involved in musculoskeletal differentiation^7^ (Supplementary Fig. S1). Mutations in the Jagged-1 (Jag1) or Notch2 genes cause Alagille syndrome in humans, an autosomal dominant disorder with characteristic skeletal manifestations, including osteopenia and high prevalence of fractures.^8^ Dominant-positive Notch2-activating mutations cause Hajdu–Cheney syndrome, which also has a strong skeletal phenotype.^9^ Interest in the role of the Notch pathway during bone development and healing has grown dramatically over the past decade, with the majority of experimental data gathered in mice. However, mechanistic interpretation of transgenic mouse models has been controversial in the field, and is confounded by variables related to experimental protocol, cell lineage state, signal strength, and microenvironment.

Genetic deletion of Jag1 in mice is embryonic lethal,^10^ and canonical RBPjκ-mediated Notch signaling regulates the activity of skeletal stem cells during development.^11^ Notch inhibition in limb bud mesenchyme (Prx1-Cre;Psen1^f/f^Psen2^-/-^ or Prx1-Cre;Notch1^-/f^Notch2^f/f^) causes stunting of the skeleton with an expanded cartilaginous growth plate, a decrease in marrow stem cell number and a reduction in osteoblast marker gene expression in vitro.^12^ Deletion of the dominant ligand, Jag1 (Prx1-Cre;Jag1^f/f^), dysregulates the osteochondral progenitor cell pool and produces compartment-specific phenotypes including femoral trabecular osteopenia in adulthood.^13^ Notch2 deletion in early osteoblasts (Runx2-Cre;Notch2^f/f^) increases bone volume (BV) in selected regions.^14^ In committed osteoblasts, Notch1 overactivation (Col1a1-Cre;N1ICD^Tg^) causes hypertrophic intramedullary osteosclerosis, and Notch inactivation (Col1a1-Cre;.Psen1^f/f^Psen2^-/-^) results in vertebral and appendicular osteopenia.^15^

In both the endochondral long bone fracture environment and during calvarial intramembranous bone healing, analyses of whole-callus mRNA and immunohistochemistry suggest that canonical Jag1/Notch2 signaling drives differentiation of post-injury mesenchyme from days five through 20.^16^ Notch blockade (Mx1-Cre;dnMAML^f/-^) alters fracture callus remodeling by day 20 but does not impair early callus formation.^17^ However, Notch-related mRNA is downregulated at days two and six post-fracture in αSMA + progenitor cells, and NICD overexpression impairs the osteogenic differentiation of this population in vitro.^18^ Pan-Notch-inactivated mesenchyme (Prx1-Cre;RBPjκ^f/f^) deposits a larger, more cartilaginous callus that never properly remodels to bone, resulting in sustained nonunion through 42 days.^19^

Taken together, there is likely a spatiotemporal switch that occurs midway through the continuum of osteoblast differentiation. Early activation of Notch drives progenitor cell proliferation and inhibits entrance into the early osteochondral phase, whereas mid-to-late activation of Notch drives osteoblast differentiation and promotes the anabolic activity of committed osteoblasts. Activating Notch in primed, post-injury mesenchyme represents a potential targeted strategy to increase bone formation.

Mammalian Notch ligands require physical association with a cell/object in order to effectively signal: soluble Notch ligands are competitive antagonists to the pathway.^20^ Jag1 can be immobilized onto a scaffold or carrier to induce osteoblast differentiation, and we have previously demonstrated feasibility of this approach in vitro using human primary cell lines.^21,22^ The goal of this study was to further define the regulatory role of Notch signaling in osteoblast differentiation and intramembranous bone healing in vivo, and to explore the therapeutic utility of Jag1 to promote bone anabolism. Our hypothesis was that Jag1 would promote osteoblastogenesis of mesenchymal progenitor cells and drive bone regeneration in experimentally induced bone defects in rodents. Herein, we demonstrate that Jag1 is a safe and effective local therapeutic for bone repair. Disruption of Jag1 during bone formation reduces bone healing, and delivery of Jag1 using clinically applicable collagen sponges significantly enhances bone healing without excess ectopic bone.

## Results

### Jag1 expression occurs concurrently with increased osteoblast differentiation genes during intramembranous bone formation

**Fig. 1:**
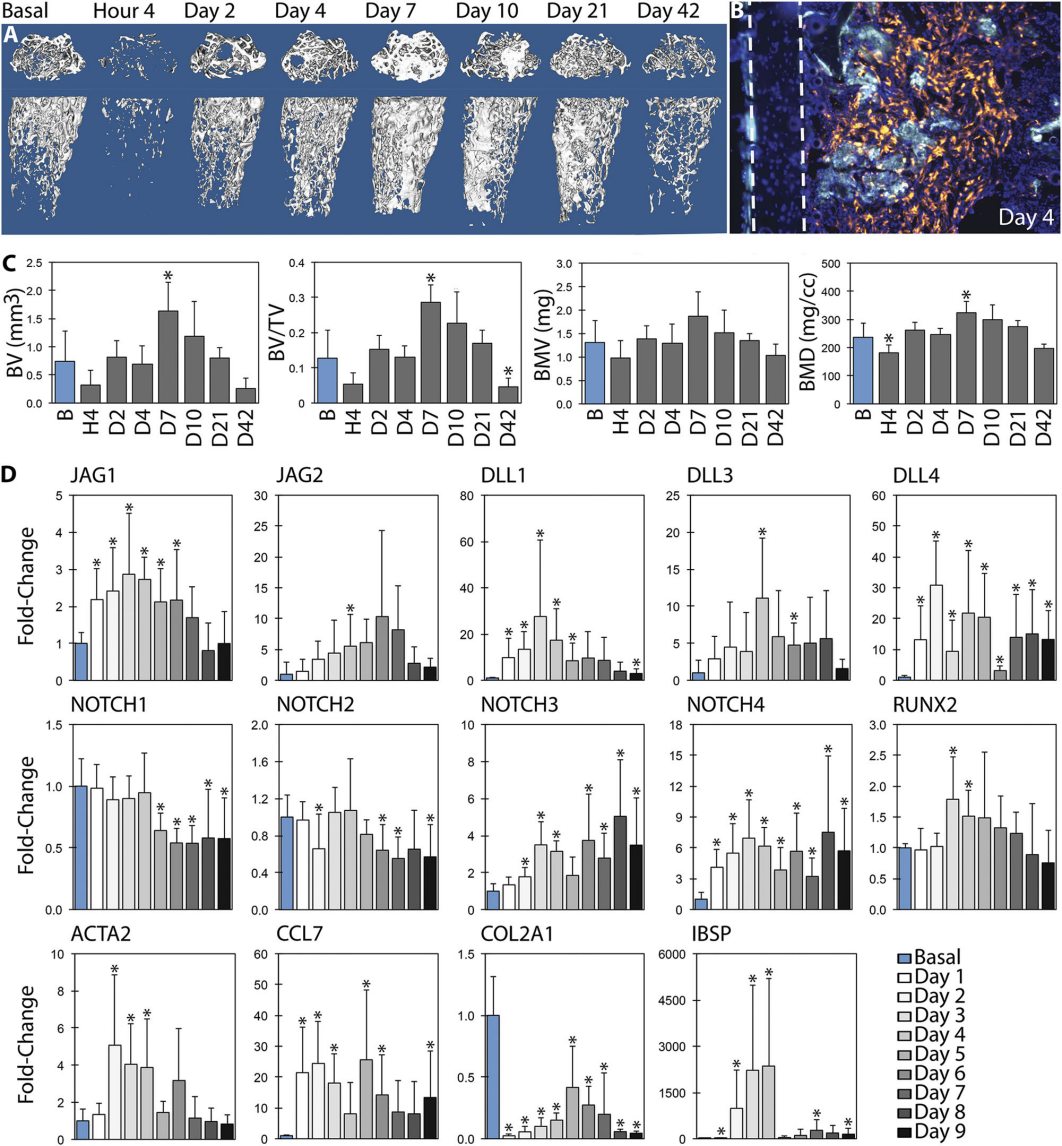
µCT and qPCR profile post-marrow ablation. Mechanical disruption of the marrow environment results in deposition of intramedullary bone within seven days. This was quantified by µCT (*n* = 6), with representative sagittal and coronal 3D reconstructions shown (**a**,** c**). Osteoblasts derived from αSMA-positive mesenchymal progenitor cells produce new (double-labeled) bone: the cortical wall is demarcated with dashed lines (**b**). Expression of Notch ligands, receptors, and select marker genes are presented over time by qPCR with respect to basal bone, relative to ACTB (**d**). Notch ligands are upregulated during osteoblast differentiation. Statistical significance is symbolized with * for *p* < 0.05 (*n* = 6–12)

Our previous work has shown that Jag1 initiated canonical Notch signaling is osteoanabolic in vitro using human mesenchymal stem cells,^21,22^ and that Jag1 disruption in vivo in osteoblast lineage cells decreases femoral trabecular bone.^13^ To more fully probe the role of Jag1 and Notch signaling in adult intramembranous bone formation, we used marrow ablation as a model that results in pronounced compartmentalized intramembranous bone formation. Marrow ablation reduced bone volume fraction (BV/TV) by 58.9% to 0.052 ± 0.033 (*p* = 0.057), followed by a spike in bone formation peaking at day 7, at which time there was 224.3% more BV/TV than basal bone (0.286 ± 0.049, *p* < 0.001), with 37.4% higher bone mineral density (BMD) (324.7 ± 38.0 mg/cc, *p* = 0.039) (Fig. 1a). Mean BV and bone mineral content (BMC) also increased. αSMA-positive mesenchymal cells and their progeny were the architects of this response, representing the dominant cell population in the regenerating region and co-localizing with fluorescent bone labels (Fig. 1b). The destruction and regeneration of intramedullary bone was followed histologically (Supplementary Fig. S2), with a blood clot forming within hours, followed by activation and proliferation of mesenchymal cells, presumably from endosteal and/or periosteal niches. The intramedullary compartment was filled with differentiating mesenchyme from days 2–4 post ablation, with the anabolic osteoblastic response peaking at day 7, followed by a catabolic phase.

In uninjured bone, JAG1 and NOTCH2 represent 95.2 and 70.3% of total marrow Notch ligand and receptor transcript, respectively (Supplementary Fig. S3). These remain the dominant Notch species throughout healing. Following marrow ablation, JAG1 was significantly upregulated at days 1–6 (Fig. 1), peaking with a 2.87 ± 1.65-fold increase in expression over basal bone at day three (*p* < 0.001). Male mice expressed a 12.0 ± 5.2% greater fold-change in JAG1 versus females at this timepoint (*p* = 0.013). Peak fold-change values for JAG2, DLL1, DLL3, and DLL4 occurred at days 6 (NS), 3 (*p* = 0.005), 4 (*p* = 0.004) and 2 (*p* < 0.001), respectively. Males exhibited statistically lower DLL1 expression (*p* = 0.002), while there were no sex differences in peak levels of JAG2, DLL3 or DLL4. NOTCH1 and 2 were downregulated and NOTCH3 and 4 were upregulated during the later stages of bone formation, with the greatest fold-change observed in the least abundant receptor, NOTCH4. Fold-change of NOTCH1 and 2 expression was 7.30–23.1% lower in males than females from days 7–9 (*p* = 0.002–0.047).

Expression of RUNX2, an essential osteoblast transcription factor, peaked at day 3 with 1.79 ± 0.69-fold expression (*p* = 0.002) and 10.4 ± 5.7% more expression in males than females (*p* = 0.018). ACTA2 (αSMA) expression, indicative of mesenchymal progenitor activation, peaked at day 2 with 5.08 ± 3.79-fold expression (*p* = 0.002) and 21.6 ± 4.2% more expression in males than females (*p* = 0.007). There was sustained upregulation of the inflammatory marker CCL7 (*p* = 0.002) and downregulation of the chondrogenic gene COL2A1 (*p* < 0.001) starting at day 1. IBSP expression peaked at days 3–4 (*p* < 0.001 and *p* = 0.032, respectively), with greater expression in females than males (*p* < 0.001). LFNG, MFNG and RFNG were not differentially regulated for more than one day (not shown).

### Jag1, but not Jag2, is required for peak intramembranous bone formation

**Fig. 2:**
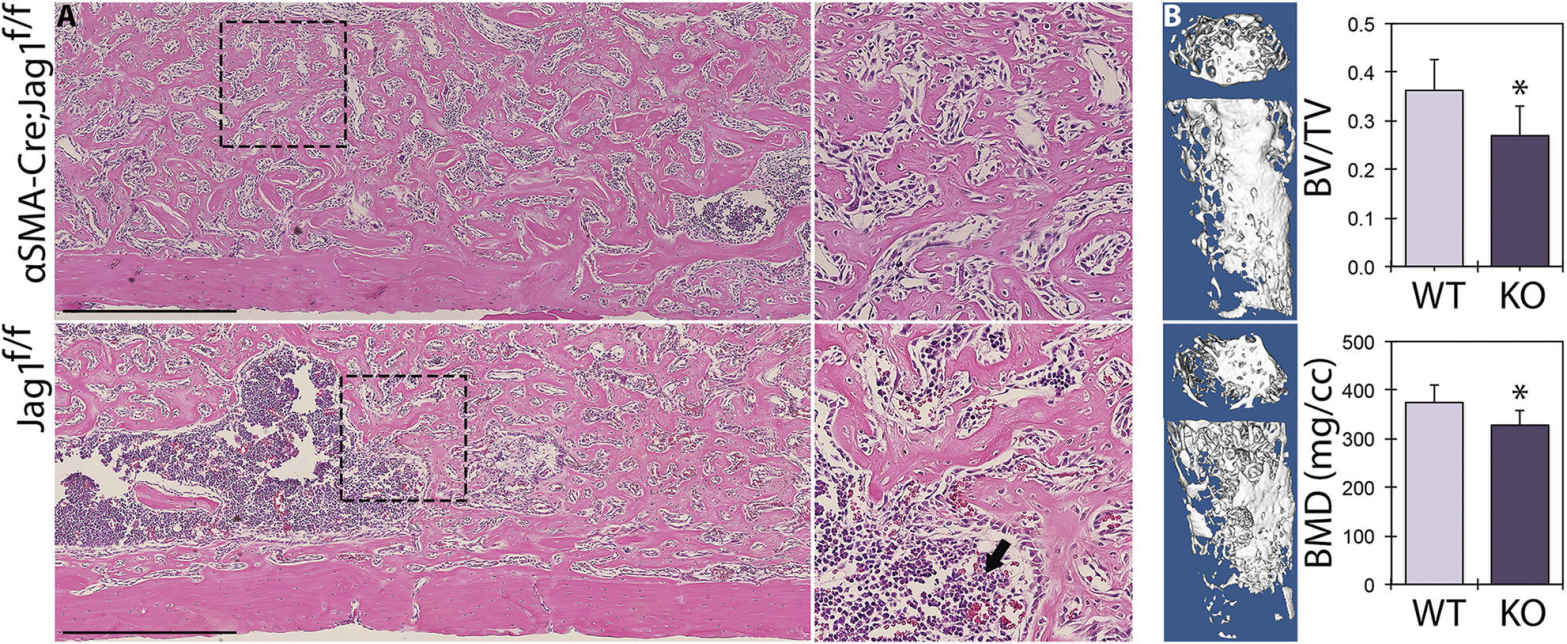
Jag1-KO reduces post-ablation bone formation. Cre-positive and Cre-negative female littermates in the Jag1^f/f^ line (*n* = 3) underwent marrow ablation procedures and received single tamoxifen injections at time of surgery. H&E histology (**a**) is shown alongside µCT reconstructions and quantitation (**b**) for day 7 post ablation. Jag1-KO animals underwent incomplete osteoblastogenesis and contain regions of marrow interspersed within the newly formed trabecular bone (arrow). Scale bars are equal to 500 µm. Statistical significance is symbolized with * for *p* < 0.05

To interrogate the role of Jag1 during intramembranous bone regeneration, we disrupted Jag1 in the early mesenchyme using inducible Cre-mediated recombination of the floxed Jag1 gene (loxP sites flanking exons 4–5), with Cre driven from the αSMA promoter (Fig. 1b). Jag1 knockout in αSMA-expressing cells at the time of bone injury (αSMA-Cre^ERT2^;Jag1^f/f^) resulted in a 25.7% reduction in BV/TV to 0.269 ± 0.056 (*p* = 0.021) and a 12.4% reduction in BMD to 327.9 ± 29.9 mg/cc (*p* = 0.032) at time of peak bone mass in day 7 females, relative to tamoxifen-treated, Cre-positive controls with wild-type Jag1 alleles (Fig. 2). Jag2 knockout (loxP sites flanking exons 1–2) using the same inducible Cre did not result in changes in BMD (*p* = 0.276) or BV/TV (*p* = 0.403) (Supplementary Fig. S4). Histologically, Jag1 knockout mice developed a dense network of intramedullary trabeculae by day 7, but the intramedullary compartment contained cavities of marrow and regions of incomplete mineralization relative to their Cre-negative counterparts (Fig. 2).

### Recombinant Jag1 improves healing of craniofacial and long bone defects in mice

**Fig. 3:**
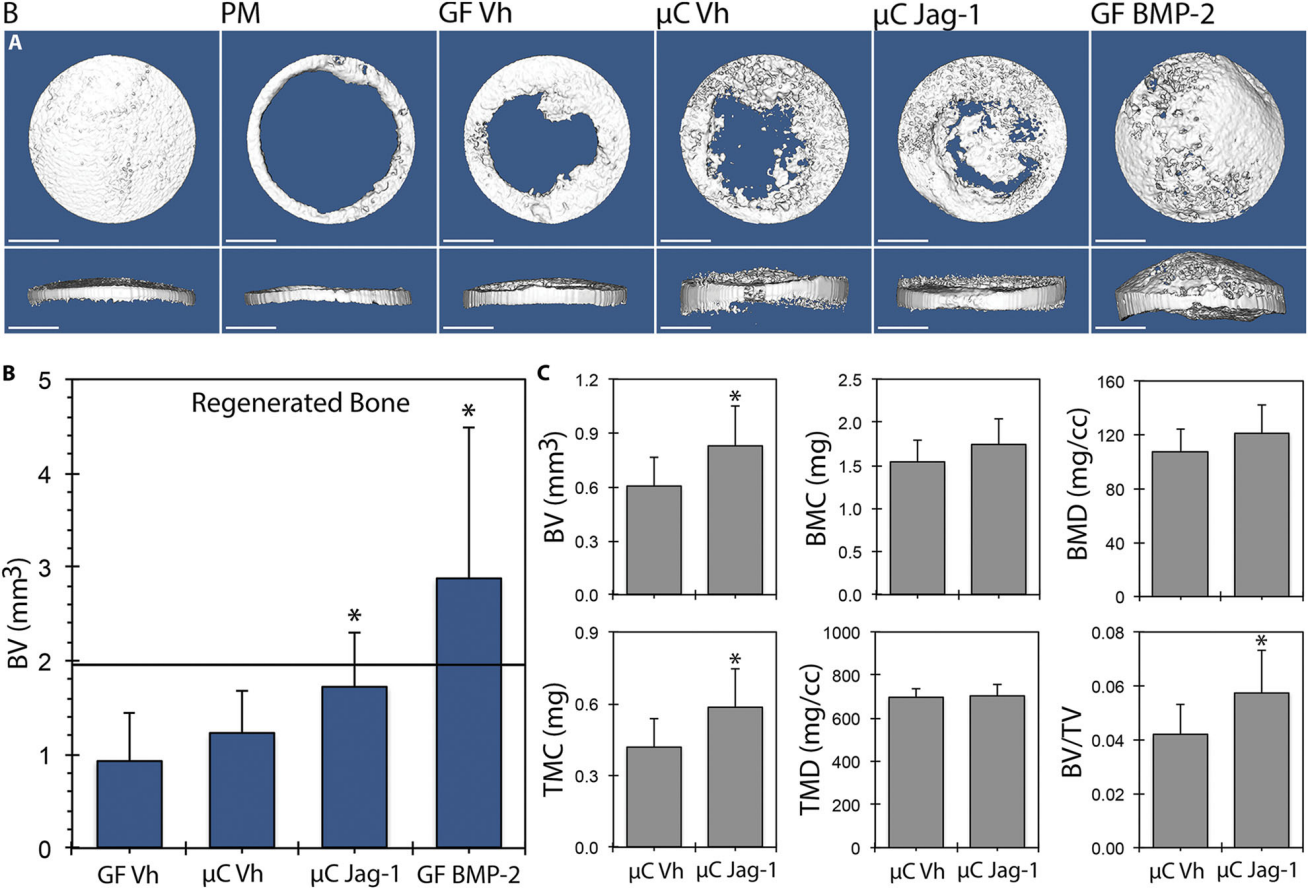
Jag1 improves healing of calvarial defects in mice. 3D reconstructions (**a**) and total regenerated bone (**b**) (PM-subtracted) were assessed using µCT 42 days post-defect, reported at a threshold of 666. Total ROI quantitation between µC-Vh and µC-Jag1 groups (**c**) was conducted at a threshold of 1650. Statistical significance is symbolized with * for *p* < 0.05 (*n* = 8–12)

**Fig. 4:**
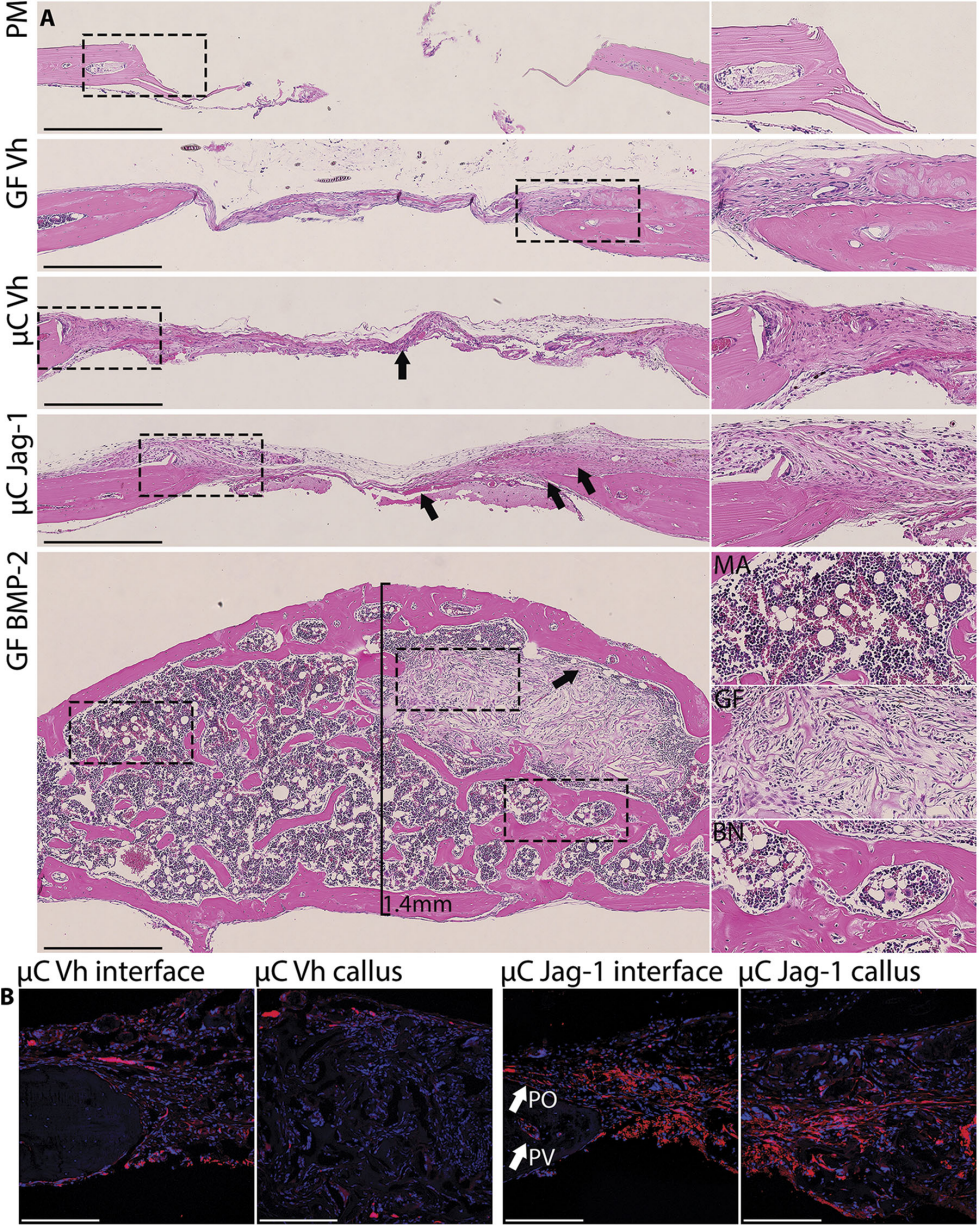
Jag1 activates αSMA-positive mesenchyme in the calvaria without bone overgrowth. Representative H&E histology (**a**) shows increased periosteal mesenchyme and improved bone matrix production 42 days post defect in the µC-Jag1 group (arrows) relative to the incomplete mineralization and fibrosis in the associated µC-Vh control (arrow). BMP2 induced supraphysiological production of bone (BN) and fatty marrow (MA), encapsulating and preserving the Gelfoam scaffold (GF). Select ROIs are zoomed adjacent to source images and marked using dashed lines. Scale bars are equal to 500 µm. αSMA-Cre^ERT2^;tdTomato reporter activity is increased in the µC-Jag1 group relative to its µC-Vh control (**b**). Confocal scale bars are equal to 200 µm

Our previous in vitro research has shown that recombinant Jag1 is an osteoanabolic factor:^21,22^ thus, we examined whether Jag1 could promote bone regeneration in vivo. Delivery of Jag1 protein to calvarial defects in mice increased bone regeneration. Representative µCT isosurfaces demonstrated new bone induced by treatment with Jag1 (µC-Jag1) or BMP2 (GF-BMP2) versus microcarrier/Gelfoam (µC-Vh) and Gelfoam (GF-Vh) controls (Fig. 3). GF-Vh grafting resulted in 0.934 ± 0.513 mm^3^ of new bone at day 42, compared with 1.222 ± 0.463 mm^3^ for µC-Vh, 1.722 ± 0.586 mm^3^ for µC-Jag1 and 2.876 ± 1.603 mm^3^ for GF-BMP2. Relative to the corresponding µC-Vh control, within the entire 3.3 × 3.3 × 1.7 mm cylindrical region of interest (ROI) surrounding the defect, the µC-Jag1 group developed 37.5% more BV to 0.828 ± 0.222 mm^3^ (*p* = 0.008), 39.8% more tissue mineral content to 0.586 ± 0.163 mg (*p* = 0.007) and 37.4% greater BV/TV to 0.0576 ± 0.0154 (*p* = 0.008). There were near-significant increases in BMC (*p* = 0.077) and BMD (*p* = 0.079). Tissue mineral density was not changed (*p* = 0.677). Importantly, the new bone formed in the µC-Jag1 group remained in the plane of the defect (Fig. 4), while in contrast, BMP2 treatment caused production of new bone that extended well above the injury margin. Marrow (with adipose) and encapsulated intact Gelfoam were observed within the regenerated bone only in the GF-BMP2 group. αSMA-positive mesenchymal cells were labeled at the time of treatment by administering tamoxifen to αSMA-Cre^ERT2^;tdTomato mice. There was a greater quantity of αSMA-positive mesenchymal cells and their progeny in the µC-Jag1 group versus the µC-Vh group, showing significantly increased osteoprogenitor cell activity. The origin and lineage of these cells will be the topic of future investigation.

**Fig. 5:**
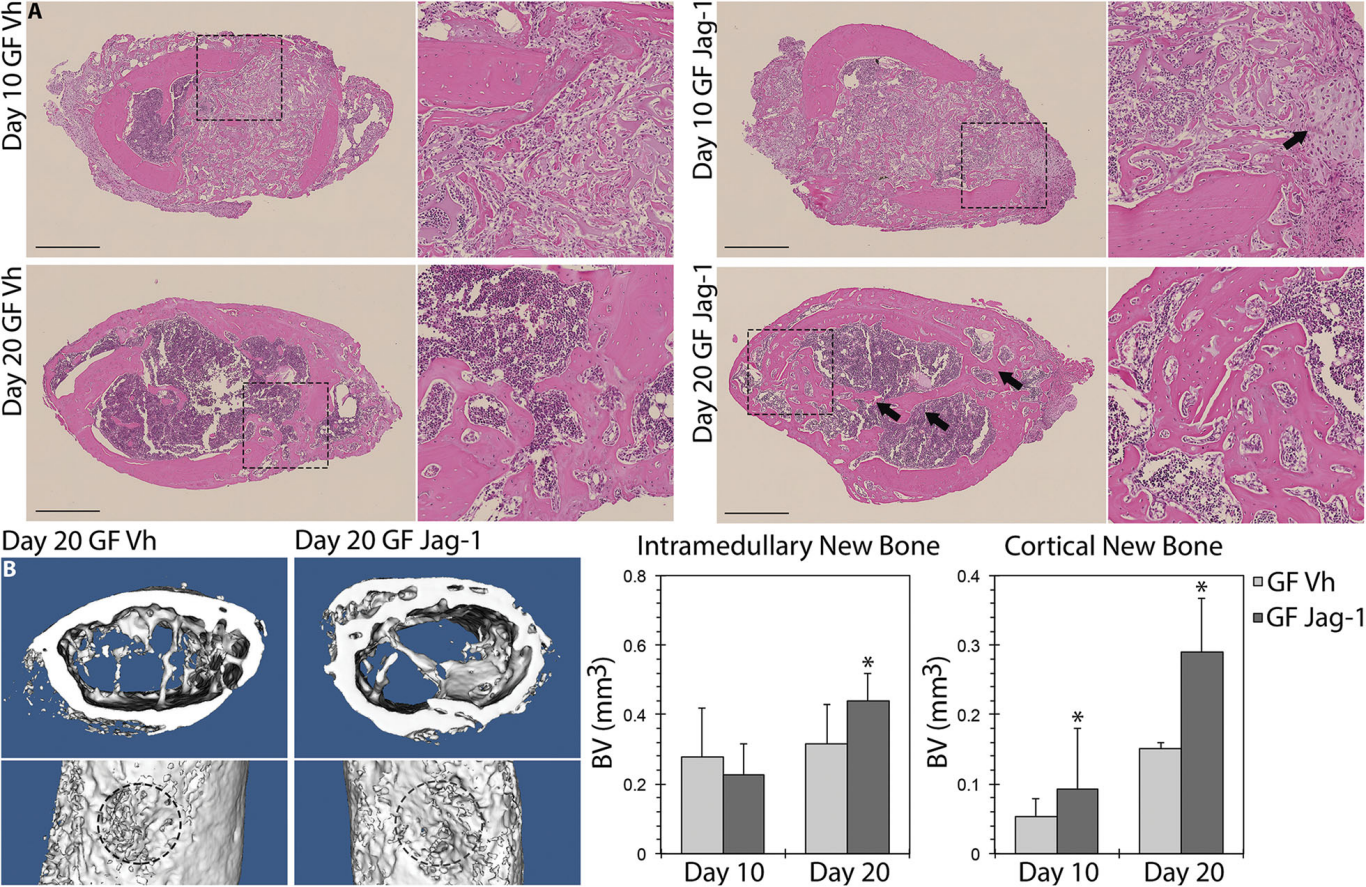
Jag1 promotes healing of femoral defects. Representative H&E histology (**a**) shows improved healing of Jag1-treated defects relative to contralateral GF-Vh controls. Selected ROIs are zoomed adjacent to source images and marked using dashed lines. At day 10, local regions of endochondral ossification occur adjacent to residual Gelfoam and the cortical margin (arrow). The Jag1-induced increase in intramedullary and cortical bone is evident at day 20 (arrows). Scale bars are equal to 500 µm. µCT isosurfaces and data **b** were gathered at a threshold of 2667. Statistical significance is symbolized with * for *p* < 0.05 (*n* = 5). Representative images represent matched sets from individual animals

Next, to determine whether Jag1 can also increase bone regeneration in the appendicular skeleton, bicortical bone defects where made in mouse femora and plugged with Gelfoam grafts bound with Jag1 (GF-Jag1) or control (GF-Vh) and examined at two timepoints. A day 10, Jag1 had induced a 71.7% increase in cortical bone relative to GF-Vh to 0.092 ± 0.007 mm^3^ (*p* = 0.024). At day 20, this difference was increased to 91.1%, 0.290 ± 0.062 mm^3^ (*p* = 0.009). There was a 40.0% increase in intramedullary bone at day 20 to 0.441 ± 0.077 mm^3^ (*p* = 0.042) (Fig. 5). The GF-Jag1 group showed pronounced periosteal activation and increased new bone relative to contralateral GF-Vh controls.

### Jag1 heals critical-sized calvarial defects in rats

**Fig. 6:**
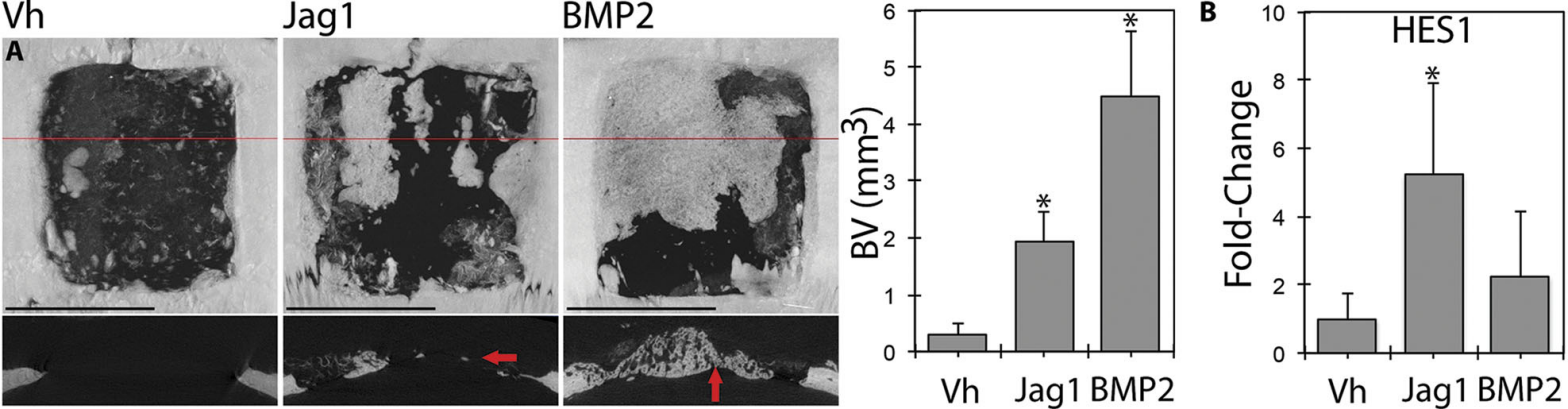
Jag1 regenerates critical-sized calvarial defects in rats. Jag1 drives pronounced bone healing in rat segmental skull defects, as seen via µCT (**a**). Representative dorsal 3D reconstructions are shown with 2D transverse sections (red lines) below. Jag1-induced healing occurs within the plane of surrounding bone (arrow), while BMP2 causes bone overgrowth above the injury margin: termed hypertrophy (arrow). Expression of the canonical Notch target gene, HES1, is increased in a frontal quadrant of rat calveriae relative to GAPDH in the Jag1-treated animals preceding this bone formation in the rostral portion of the healing defect (**b**). Associated quantitation represents bone within 6.6 × 6.6 mm ROIs centered in each defect. Scale bars are equal to 5 mm. Statistical significance is symbolized with * for *p* < 0.05 (*n* = 4–6)

Finally, we used a rat non-healing critical size defect to explore whether Jag1 could promote regeneration of non-healing injuries in a species other than mouse. µCT analysis of large non-healing calvarial defects in rats demonstrates the profound healing potential of Jag1 (Fig. 6). Sixty days following surgery, total BV was 0.286 ± 0.194 mm^3^ in the Vh group, 1.922 ± 0.537 mm^3^ in the Jag1 group (671% of Vh, *p* < 0.001) and 4.471 ± 1.154 mm^3^ in the BMP2 group (1560% of Vh, *p* < 0.001). Again, similar to observations with BMP2 in mice, BMP2 in rats resulted in hypertrophic bone extending above the margins of the surrounding bone, while Jag1 produced more regular bone that was within the plane of the original calvaria. Expression of the basic helix-loop-helix transcription factor HES1, a canonical Notch target gene, was increased 5.24 ± 2.66-fold at day 5 (*p* = 0.010), demonstrating that Jag1 was functionally signaling.

## Discussion

There is an urgent need to develop new therapeutics for bone repair. While BMPs have proven to be potent tools and remain the clinical standard for promoting bone regeneration, over the past 5 years it has become clear that BMPs are not universally beneficial. Potential adverse effects of BMPs include inflammation, adipogenesis, ectopic/hypertrophic bone, radiculopathy and death.^23^ Additionally, BMPs can induce osteoclastogenesis in individuals with osteoporosis, exacerbating loss of mineral content and disqualifying a significant patient population. We demonstrate that the Notch signaling ligand Jag1 is required for maximal intramembranous bone regeneration and that delivery of Jag1 can promote new bone healing in both rats and mice.

In a marrow ablation model, which offers a method of studying intramembranous bone formation within a discrete physiological compartment on a condensed timeline,^24^ Notch signaling elements, particularly Jag1, were transcriptionally correlated with the differentiation of αSMA-positive mesenchyme to osteoblasts (COL2A1-negative, cartilage-free). In this model, cells transition from ACTA2-expressing progenitor cells (day 2) through RUNX2-positive pre-osteoblasts (day 3) into IBSP-positive anabolic osteoblasts (days 3–4), which then rapidly fill the marrow cavity with bone.^25^ These mouse data corroborate a published microarray in rats, in which Jag1 was the most abundant ligand (upregulated 2.54-fold at day 5 post-ablation), Notch2 was the most abundant receptor and DLL4 showed the greatest increase in expression (5.97-fold).^26^ Perturbing Notch via tamoxifen-inducible homozygous deletion of Jag1 in that mesenchymal lineage at the time of healing (αSMA-Cre^ERT2^;Jag1^f/f^), but not Jag2 (αSMA-Cre^ERT2^;Jag2^f/f^), resulted in reduced bone regeneration.

Having identified a positive regulatory role of Jag1 in adult bone healing, we sought to therapeutically deliver Jag1 protein to the site of a calvarial defect in mice, which heals by intramembranous ossification. Notch (DLL4) gain-of-function has been proposed as a therapeutic paradigm for muscle healing,^27^ but recombinant Notch ligand has, until now, never been used to drive regeneration. Agarose Protein G beads were previously used to deliver Jag1 in vitro,^22^ so a similar approach was adopted using 5μm-diameter biodegradable PLGA microcarrier beads (rigid substrate for Jag1) embedded in Gelfoam (osteoconductive scaffold). This approach resulted in significantly greater defect coverage and BV versus microcarrier-laden vehicle by µCT 42-days post-defect. The positive control, BMP2, delivered at an approximately equimolar concentration, produced a complex boney mass, with retained Gelfoam and adipocyte-laden marrow, protruding well above the surrounding parietal bones. The µC-Jag1 group had 59.9% of the regenerated BV of the GF-BMP2 group, but importantly, the new bone remained in the plane of the defect, and was qualitatively more similar to the native cranial bone.

Overproduction of bone by BMPs, including potentially fatal ectopic bone and malignant transformation,^23^ is a clinical problem that we hope to overcome with targeted delivery of Notch ligands. While delivered in equimolar doses here, the high potency of BMP2 relative to Jag1 suggests that fine-tuning dose will be an important aspect of promoting a physiologically appropriate bone anabolic response. BMPs are osteoinductive in solution and migrate away from the application site. Unlike BMP2, Jag1 appears to promote a bone-anabolic phenotype only among cell types that are predisposed to form bone, such as post-injury mesenchyme: enhancing the safety profile. We hypothesize that Jag1 treatment will be broadly therapeutic, though ALGS patients, who are predisposed to pathologic femur fractures that heal poorly, likely stand to benefit more. Furthermore, collagen matrices including Gelfoam are also available in injectable form, increasing applicability of Jag1-functionalized scaffolds beyond those injuries requiring open reduction. Long-term safety studies of Notch-activating therapies in bone will be required to corroborate these hypotheses.

To demonstrate applicability of Jag1 therapy to the appendicular skeleton using a simpler clinical delivery methodology, Jag1 was adhered directly to Gelfoam (microcarrier-free) and then administered to the site of a bicortical window defect in the femur. This model is intended to be intramembranous, but does include some regions of endochondral ossification near the periosteal margins of the defects. Jag1 enhanced cortical and intramedullary ossification in this context. The most profound healing was observed in the largest, most clinically relevant injury, when Jag1 was delivered to critical, non-healing calvarial defects on Helistat sponges in rats. Mirroring the mouse data, the Jag1 group resulted in 43.0% of the regenerated BV of the BMP2 group, 671% more than vehicle control, but the new bone was formed inline with the native calvarial bone, without the hypertrophic phenotype. HES1 was upregulated in the Jag1 group, confirming the bioactivity of the recombinant protein via canonical Notch signaling in this model.

In future experiments it will be important to define the mechanistic basis of this improved healing. While results from our laboratory and others show that Jag1 and canonical Notch signaling can direct osteoblast differentiation in a homotypic manner,^13^ it is feasible that the effect of Jag1 extends beyond the mesenchymal lineage. Notch signaling regulates elements of vascular architecture that are essential for angiogenesis and the osteoprogenitor niche.^28^ Ectopic Jag1 can cause a primary vascular phenotype, in addition to altered mesenchymal-endothelial crosstalk,^29^ which were not investigated in this study. Furthermore, with autografts functioning as the current standard of care for bone replacement, it will be important to characterize the osteoinductive efficacy of Jag1 on structural, load-bearing materials:^30^ particularly due to the mechanosensitivity of ligand presentation on receptor activation.^31^ Finally, fracture healing is significant translational goal of our approach—it will be important to determine whether or not Jag1-mediated Notch signaling has the same osteoblastic phenotype during chondrocyte transdifferentiation during endochondral ossification.^32^

In conclusion, homotypic Jag1-mediated canonical Notch signaling within adult mesenchymal progenitor cells promotes osteoblast differentiation and bone healing. This phenomenon is conserved across the craniofacial and appendicular skeleton and from mice to rats, and the therapy is effective with two different collagen scaffolds with or without the presence of microcarrier beads. Jag1 may be functioning to increase proliferation of early-stage progenitor cells,^33^ to promote late-stage differentiation and anabolic activity of osteoblasts,^22^ or both (Supplementary Fig. S5). Direct delivery of recombinant Jag1 to bone injuries represents a therapeutic approach for skeletal regeneration.

## Materials and methods

### Study design and animal use

All research was conducted following all relevant procedures and guidelines, under the guidance and approval of the Institutional Animal Care and Use Committees of the University of Michigan, Michigan State University or the University of Pennsylvania. Wild-type C57BL/6 mice and Sprague Dawley rats were enrolled in the reported studies. The following transgenic mouse strains on a C57BL/6 background were also used: αSMA-Cre^ERT2^;tdTomato,^25^ Jag1^f/f^,^34^ αSMA-Cre^ERT2^; Jag1^f/f^, Jag2^f/f^,^35^ αSMA-Cre^ERT2^;Jag2^f/f^. Recombination was induced by a single intraperitoneal injection of 75 mg/kg tamoxifen at time of surgery. Animals were socially housed when possible and allowed ad libitum access to food and water. Isoflurane gas (Abbott Labs) was used for inducing and maintaining surgical anesthesia under external heat and animals received subcutaneous 1 mg/kg Buprenorphine SR (ZooPharm) for post-operative analgesia. Incision sites were cleared of hair and disinfected with alternating scrubs of alcohol and povidone–iodine. Euthanasia was performed in accordance with current American Veterinary Medical Association guidelines. Surgeries and quantitative analyses were conducted with investigators blinded from timepoint, genotype and treatment.

### Mouse femoral marrow ablation surgery

Stifles were clipped and scrubbed and longitudinal incisions at the patella were created using sterile surgical instruments, exposing the intercondylar area of the femurs. 25-gauge needles were manually drilled through the intermedullary canal toward the proximal growth plate and repeatedly reamed. 26-gauge needles were then used to flush the cavity with saline until the discharge was clear. Incisions were closed with surgical glue. For gene expression analysis, 6–12 animals were used per time point, with each time point representing a 1:1 ratio of males to females. Six animals per timepoint (*n* = 3 males, *n* = 3 females) were used for μCT analysis and histology. Four animals were used for fluorescence double-labeling and reporter activity. Mice in this experiment were 19 ± 5 weeks old and weighed 26 ± 4 g. For knockout data and associated controls, *n* = 3 females mice were used per group, at 10.0 ± 1.1 weeks old and 20.7 ± 1.1 g.

### Mouse calvarial defect and grafting

Absorbable gelatin sponges (Pfizer Gelfoam 12–7 mm, as above) were sliced longitudinally (1 mm thick) and cut into discs using a 4-mm biopsy punch (Integra 33–34). Collagen grafts were pre-hydrated in phosphate-buffered saline (PBS) overnight, expunged of fluid using a cotton tipped applicator (Puritan 806-WCL), placed into round bottom 96-well plates and hydrated with PBS (vehicle), 25 μg of 5 μm-diameter green fluorescent PLGA microcarrier beads (Phosphorex Degradex) in PBS (microcarrier control), 10 μL of 1.25 mg/mL recombinant human Jag1 (R&D Systems 1277-JG) on 25 μg microcarrier beads in PBS or 0.125 mg/mL recombinant human/mouse BMP-2 (R&D Systems 355-BM) in 800 nM HCl with 0.02% FBS in PBS two hours before the start of surgeries and incubated at 37 °C. Recipient mice in this study were 14 ± 3 weeks old and weighed 25 ± 4 g.

Parietal bones were exposed by single midline sagittal incisions and scraped of periosteum over the defect sites to facilitate drilling. Bilateral calvarial defects were then created using a Piezosurgery GP (Mectron) piezoelectric bone drill with an (3 mm-diameter) OT11 osteoplasty/osteotomy insert with external saline irrigation, resulting in clean holes free of thermal damage with intact meninges. These critical size defects were immediately repaired using a graft from a single experimental group. All defects were produced by one author (D.W.Y.), all grafts were placed by another author (R.S.), and both of these individuals were blinded from the assigned treatment at time of surgery. Incisions were closed with surgical glue and animals were closely monitored during recovery.

### Mouse femoral defect and grafting

US FDA-approved absorbable gelatin sponges (Pfizer Gelfoam 12–7 mm), 2 mm thick and cut using a 1.5 mm biopsy punch, were incubated for 45 min prior to the start of surgeries in 15 μL of either PBS (vehicle) or 400 μg/mL recombinant rat Jag1 (R&D Systems AF599) in PBS. The surgical sites were accessed by craniolateral incision and blunt dissection between the Rectus femoris and Vastus lateralis muscles. Femurs were elevated using forceps and through-and-through window defects were created in the distal metaphysis 5 mm proximal to the femoral trochleae using a Dremel 8100 with a 0.75 mm drill bit. Defects were plugged with collagen grafts from each group (left side vehicle, right side Jag1) and incisions were sutured shut. Ten animals received bilateral femoral window defects, with *n* = 5 analyzed at day 10 and *n* = 5 analyzed at day 20.

### Rat calvarial defect and grafting

Sprague Dawley rats received 7.0 × 7.0 mm square critical-sized calvarial defects centered on the sagittal suture, using a piezoelectric bone drill with OP7 (periosteum) and OTS (osteotomy) inserts. 1.1 × 1.1 × 0.5 cm absorbable collagen hemostatic sponge grafts (Helistat, Integra Life Sciences Corporation) were functionalized with PBS (vehicle), 50 µg of recombinant human Jag1 or 5 µg of recombinant human/mouse BMP-2 and placed within the defects. Incisions were closed with 4-0 nylon suture. Fourteen animals were harvested at 60 days and 12 animals were harvested at 5 days. Rats weighed 450–550 g and were 5–6 months of age.

### Sample preparation and histological analysis

Tissues were harvested and fixed in 10% buffered formalin under agitation for 72 h. Samples destined for fluorescence microscopy were then transferred to 30% sucrose in PBS under agitation for 48 h and then stored at 4 °C. Samples destined for μCT and histology were transferred to 70% ethanol and stored at 4 °C. After μCT imaging, these bones underwent routine processing and embedding in paraffin, with hematoxylin and eosin slides prepared by the histology cores of Michigan State University or the University of Michigan Dental School. Slides were imaged on a Nikon Eclipse Ni upright microscope with DS-Ri2 camera and Prior OptiScan II motorized stage, scanned and stitched at 20× in MIS Elements BR software. Basic post-processing and figure design were conducted in Adobe Photoshop CS6.

### Micro-computed tomography (μCT)

Ablated mouse femurs and mouse calveriae were scanned in GE Healthcare eXplore Locus instruments. Marrow-ablated femurs and calveriae were scanned in water at 18 µm voxel size using the following settings: 80 kV, 80 µA, 1600 ms, 400 views. Reconstruction and analysis was performed in MicroView (Parallax Innovations). The intramedullary ROI for each ablated femur was manually defined, consisting of 25% of the total femur length, shifted 5% proximal to the distal growth plate, at a threshold of 1650. For mouse calvarial samples, BV contained within 3.33 × 3.33 × 1.66 mm ROIs centered over each defect were quantified. Representative images were created using smoothened isosurfaces at these settings. For calvarial grafts, new BV was calculated by subtracting the BV contained within the ROI following creation of a fresh defect post-mortem (PM), such that no healing had occurred. A low threshold of 666 was used here for comparing treatments with different-density bone. Comparisons between the µC-Vh and µC-Jag1 groups were further elaborated at the standard, more conservative threshold of 1650 using total quantitation within the ROI without subtraction of PM so as to allow for quantitation of density.

Mouse tibial defects were scanned in a Scanco µCT 35 at 21 µm voxel size using the following settings: 55 kV energy, 145μA intensity, 650 ms, 500 views. User-defined contours with automated interpolation were used to define ROIs encompassing cortical bone, the defect and associated callus. Similarly, user-defined contours were drawn around the original cortical bone for exclusion. This semi-automated segmentation method analyzes the callus outside the pre-existing cortical bone. The callus was analyzed in Scanco software using Gaussian filtering with a global mineral threshold of 260 mgHA/cm^3^.

For rat calveriae, analysis was performed using a Bruker SkyScan 1076. Specimens were immersed in water and scanned individually at 9 µm voxel size using the following settings: 65KV, 381 µA, 200 ms, 600 views. Images were reconstructed using NRecon software with attenuation coefficient 0.005534, smoothing 3.0, misalignment compensation 2.0, ring artifact reduction 10, beam-hardening correction 30%. DataViewer software was used to re-align the images and quantitative parameters were assessed using CTan software. Bone voxels were thresholded between 63–255 by averaging auto-thresholds across each file. 3D analysis was done on 6.6 × 6.6 mm area that was adjusted in the *z*-axis to encompass all new bone.

### Gene expression analysis

For marrow ablation, intramedullary tissue was harvested by clipping the proximal and distal epiphyses and repeatedly reaming/flushing the diaphysis from both ends with 1 mL of TRIzol per animal using a 22 G needle and syringe. For mouse calvarial defects, a 4-mm biopsy punch was used to collect tissue centered over each defect, which was subsequently flash-frozen in liquid nitrogen in TRIzol and homogenized in a cryogenic bead mill (Bertin Precellys). For rat calvarial defects, a frontal quadrant of each day 5 sample was processed for gene expression analysis. RNA was isolated by acid guanidinium thiocyanate-phenol-chloroform extraction with GlycoBlue coprecipitant followed by purification with Qiagen RNeasy Midi spin columns with on-column DNase digestion per the manufacturer’s protocol, then reverse-transcribed. qPCR reactions were conducted with 20ng of template using custom primers (Table S1) and SYBR Select master mix for 40 cycles and analyzed using the 2^-ddCt^ method. Fold-change data is reported as mean ± standard deviation relative to basal tissue.

### Fluorescence microscopy

Conventional fluorescence images of 7 µm-thick fixed, non-decalcified, cryosectioned femurs from αSMA-Cre^ERT2^;tdTomato marrow-ablated mice were acquired on a Nikon Eclipse 50i POL microscope. Half of the animals received tamoxifen at time of surgery; all animals received subcutaneous injections of 10 mg/kg calcein (Sigma C0875) at time of surgery and 50 mg/kg demeclocycline hydrochloride (Sigma D61400) at day three. Animals were sacrificed at day 4 and examined for label uptake and tdTomato fluorescence.

Confocal images of 9µm-thick fixed, non-decalcified, coronal cryosections of grafted calveriae were acquired on a Nikon A1 + upright confocal microscope using a CFI Plan Apochromat λ VC 20× air objective. Samples were collected from αSMA-Cre^ERT2^;tdTomato mice and slides were co-stained with DAPI. A 403 nm laser was used to excite DAPI and a 560 nm laser was used to excite tdTomato. A 405/488/561/647 main dichroic mirror was used. Emission dichroic mirrors were used to separate emission wavelengths for each detector channel. Emission from DAPI was separated and filtered using a 505LP dichroic mirror and a 450/50BP filter. Emission from tdTomato was separated and filtered using a 560LP dichroic mirror and a 595/50BP filter. A 5 slice, 7 µm, z-series confocal image was acquired and a maximum intensity projection was created, with a 1024 × 1024 frame size and 2× Kalman averaging to decrease noise.

### Statistical analysis

Data values are expressed as mean ± standard deviation. Statistical significance was determined by two-way Student’s *t*-tests versus basal, wild-type or vehicle controls. For qPCR data within the marrow ablation timecourse, suspected outliers (5% of dCt values) were screened via a two-way Dixon’s *Q*-test or a Rosner’s extreme Studentized deviate test with *p* < 0.05. No outliers were removed from other data sets. Points of significance are annotated graphically in figures with *≡*p* < 0.05.

### Data availability

The data sets generated during and/or analyzed during the current study are available from the corresponding author on reasonable request.

## Electronic supplementary material

Table S1

Figure S1

Figure S2

Figure S3

Figure S4

Figure S5

Figure S6
