# Supplementary material for: Intraoperative delivery of the Notch ligand Jagged-1 regenerates appendicular and craniofacial bone defects
Source: NPJ Regen Med. 2017 Dec 15;2:32. doi: 10.1038/s41536-017-0037-9 (PMC5732299; doi:10.1038/s41536-017-0037-9)
Supplement: Supplementary file 1 — Table S1 [file 41536_2017_37_MOESM1_ESM.docx]

| Gene | | | Forward | Reverse |
| --- | --- | --- | --- | --- |
| m | *ACTB* | Beta-actin | AAGAGCTATGAGCTGCCTGA | TGGCATAGAGGTCTTTACGG |
| m | *JAG1* | Jagged-1 | CAGTGCCTCTGTGAGACCAA | AGGGGTCAGAGAGACAAGCA |
| m | *JAG2* | Jagged-2 | GGCAAAGAATGCAAAGAAGC | TGGCTGCCACAGTAGTTCAG |
| m | *DLL1* | Delta-like 1 | TTAGCATCATTGGGGCTACC | TAAGTGTTGGGGCGATCTTC |
| m | *DLL3* | Delta-like 3 | CCAGTAGCTGCCTGAACTCC | ATTGAAGCAGGGTCCATCTG |
| m | *DLL4* | Delta-like 4 | ACCTTTGGCAATGTCTCCAC | TTGGATGATGATTTGGCTGA |
| m | *NOTCH1* | Notch 1 | CAGCTTGCACAACCAGACAGAC | ACGGAGTACGGCCCATGTT |
| m | *NOTCH2* | Notch 2 | ACAAATACTGTGCAGACCACTTCAA | AGCACCACGATGATCAGGGT |
| m | *NOTCH3* | Notch 3 | CAGGCGAAAGCGAGAACAC | GGCCATGTTCTTCATTCCCA |
| m | *NOTCH4* | Notch 4 | ACCACAATGAGTGCCTGTCA | TTGGTTCAGGCAGGGATTAG |
| m | *RUNX2* | Runt-related transcription factor 2 | CCCAGCCACCTTTACCTACA | TATGGAGTGCTGCTGGTCTG |
| m | *ACTA2* | Alpha 2 smooth muscle actin | CAGGCATGGATGGCATCAATCAC | ACTCTAGCTGTGAAGTCAGTGTCG |
| m | *CCL7* | C-C motif chemokine ligand 7 | CTCATAGCCGCTGCTTTCAGCATC | GTCTAAGTATGCTATAGCCTCCTC |
| m | *COL2A1* | Collagen type 2 alpha 1 | GGCTCCCAGAACATCACCTA | TCGGCCCTCATCTCTACATC |
| m | *IBSP* | Integrin binding sialoprotein | CTCCTCTGAAACGGTTTCCA | TTCGTTTGAAGTCTCCTCTTCC |
| m | *LFNG* | Lunatic fringe | CACAGAACGGATCAGCGAGC | TGAAGTGTCCTCCACTGGCC |
| m | *MFNG* | Manic fringe | TCTTGGTCAGTGGCCTCAGG | CACATAGACATCACGGTCCTGC |
| m | *RFNG* | Radical fringe | CCTGCTTTCCACCTTCTCTTCC | AGCCCCACCAGTAGCAAACC |
| r | *GAPDH* | Glyceraldehyde 3-phosphate dehydrogenase | ACAGTCCATGCCATCACTGCC | GCCTGCTTCACCACCTTCTTG |
| r | *HES1* | Hes family BHLH transcription factor 1 | CAACACGACACCGGACAAAC | CGGAGGTGCTTCACTGTCAT |

**Supplementary Table 1.** Primers used for mouse (m) and rat (r) qPCR with SYBR reagents.
