# Supplementary figures and images for: Intraoperative delivery of the Notch ligand Jagged-1 regenerates appendicular and craniofacial bone defects
Source: NPJ Regen Med. 2017 Dec 15;2:32. doi: 10.1038/s41536-017-0037-9 (PMC5732299; doi:10.1038/s41536-017-0037-9)

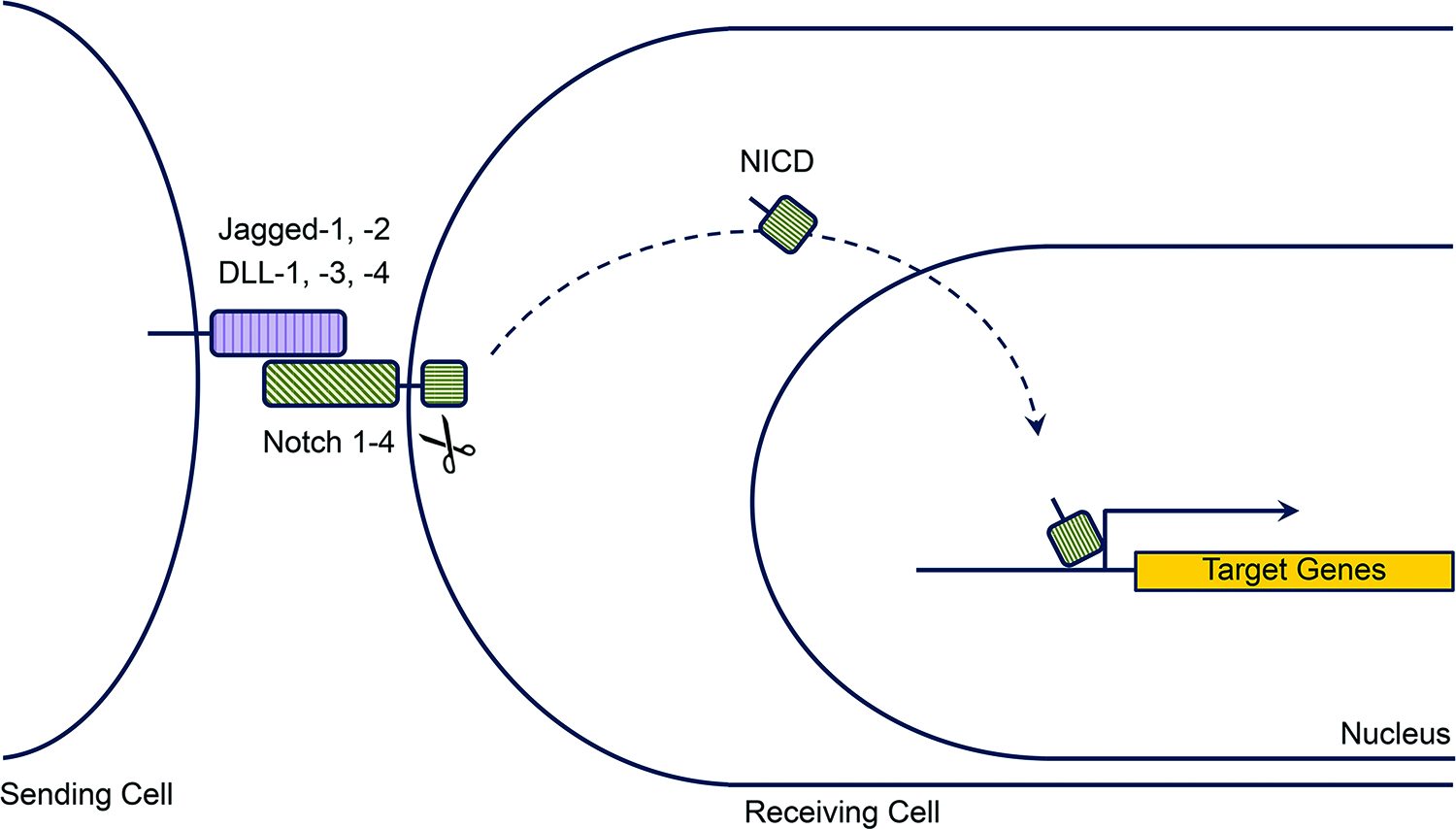

Supplement: Supplementary file 2 — Figure S1 [file 41536_2017_37_MOESM2_ESM.tif]

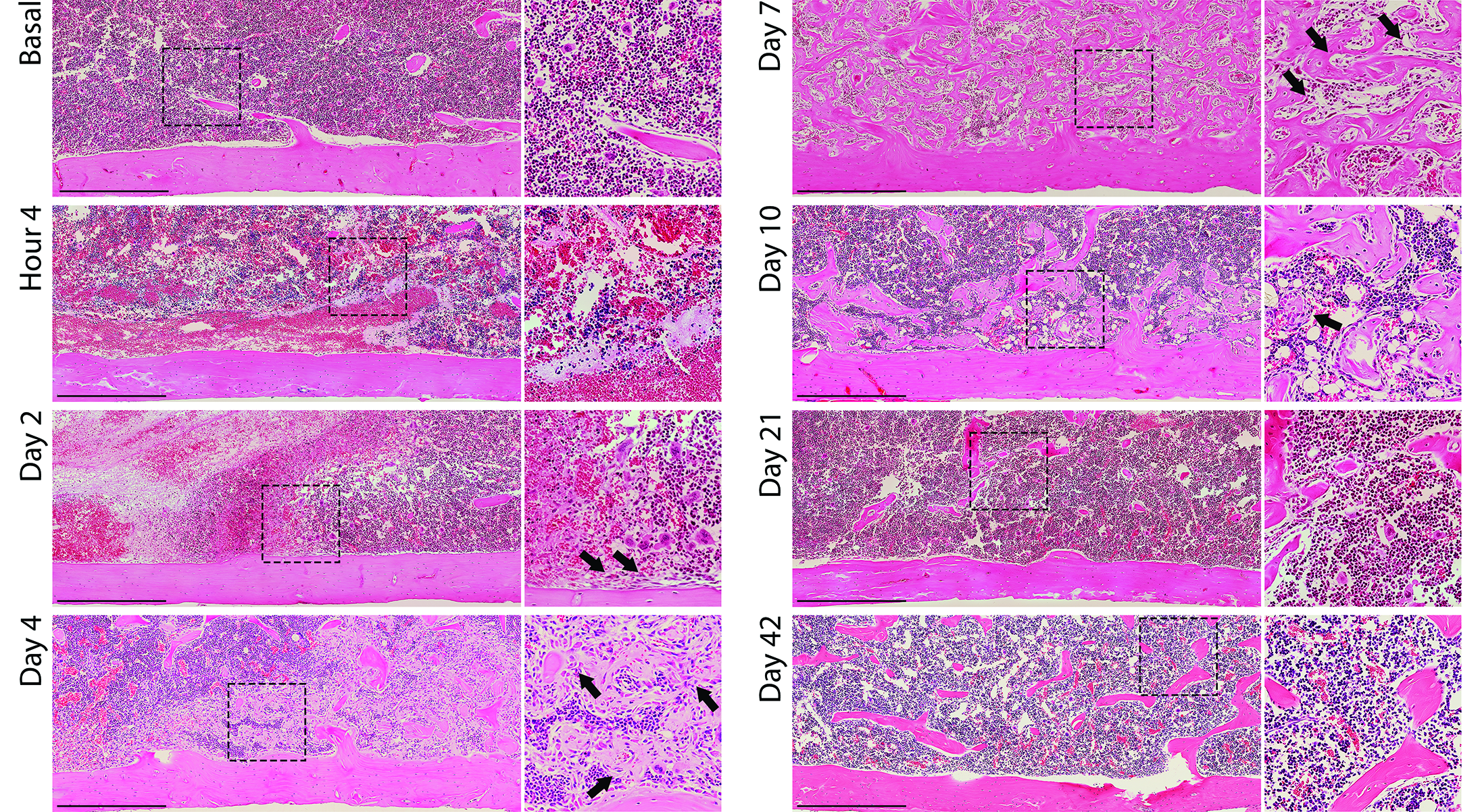

Supplement: Supplementary file 3 — Figure S2 [file 41536_2017_37_MOESM3_ESM.tif]

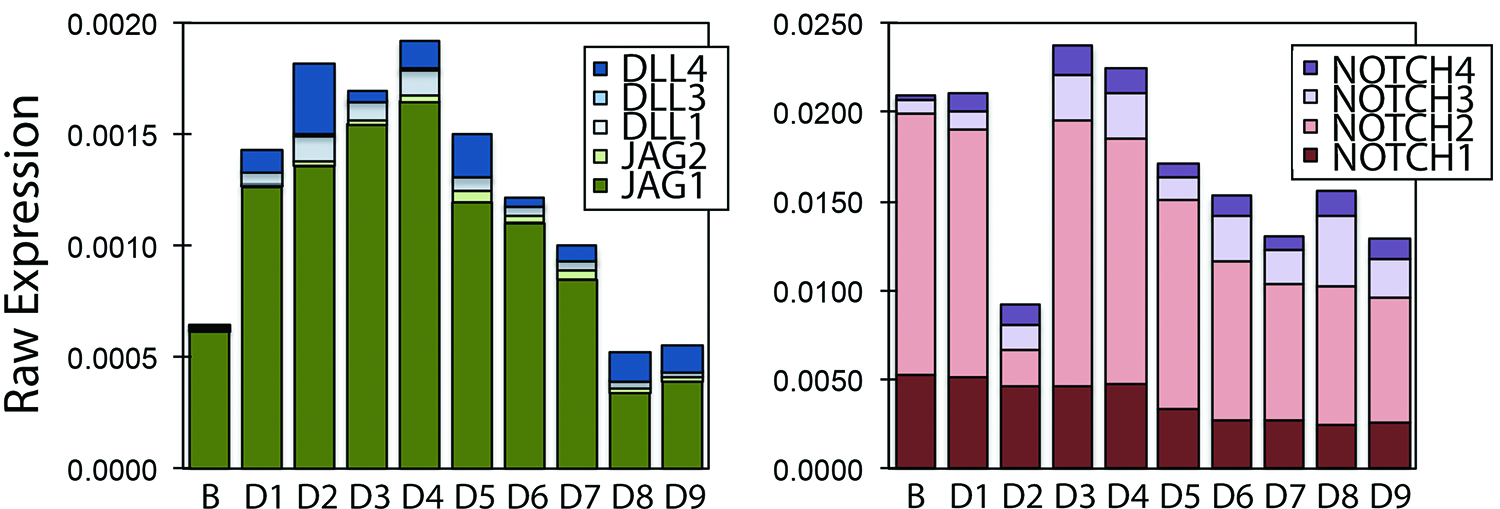

Supplement: Supplementary file 4 — Figure S3 [file 41536_2017_37_MOESM4_ESM.tif]

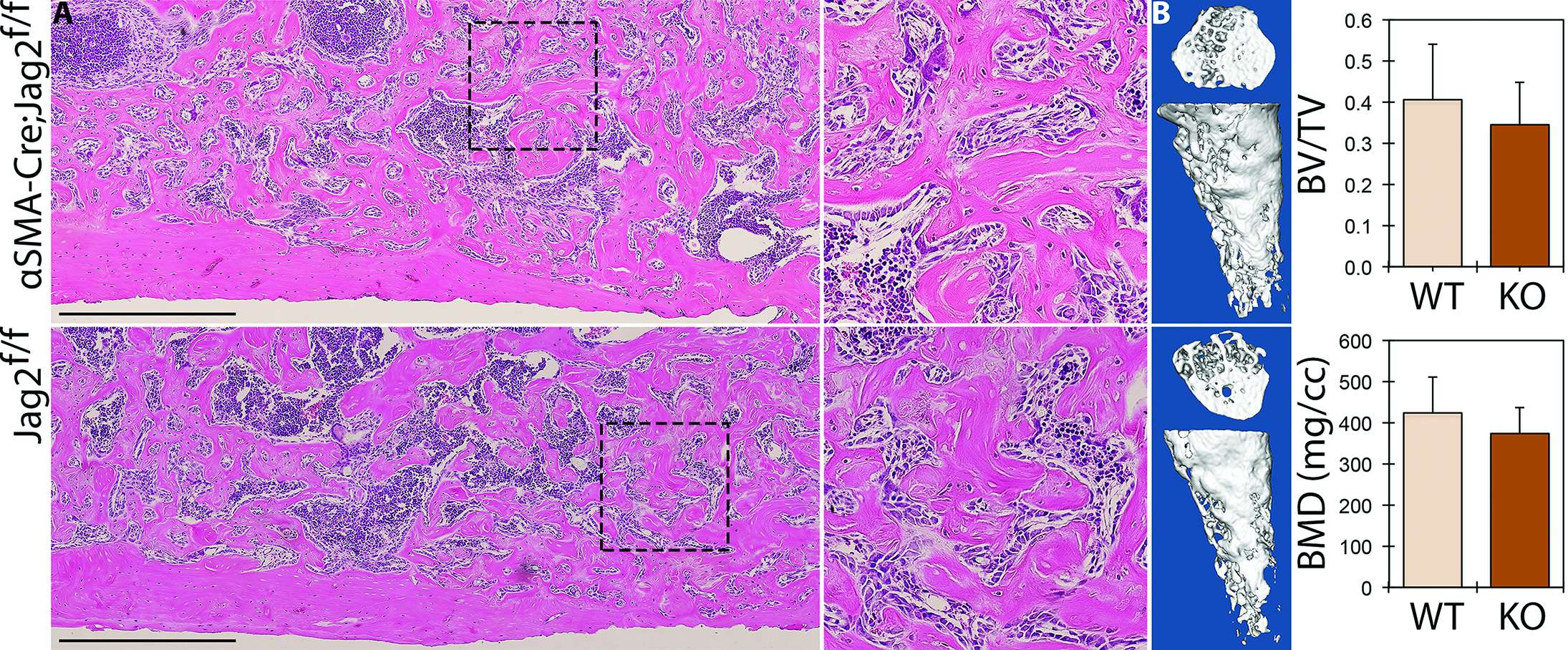

Supplement: Supplementary file 5 — Figure S4 [file 41536_2017_37_MOESM5_ESM.tif]

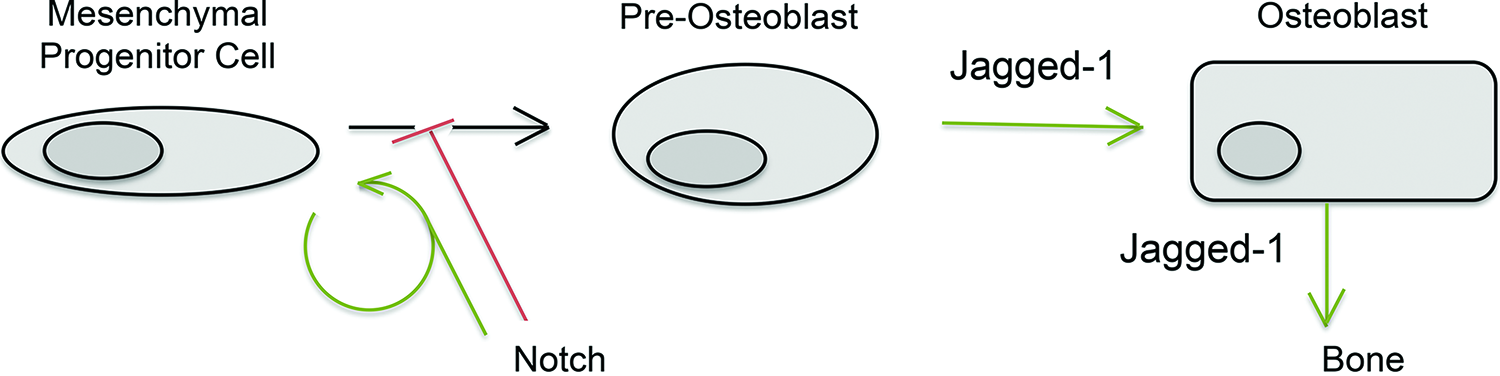

Supplement: Supplementary file 6 — Figure S5 [file 41536_2017_37_MOESM6_ESM.tif]

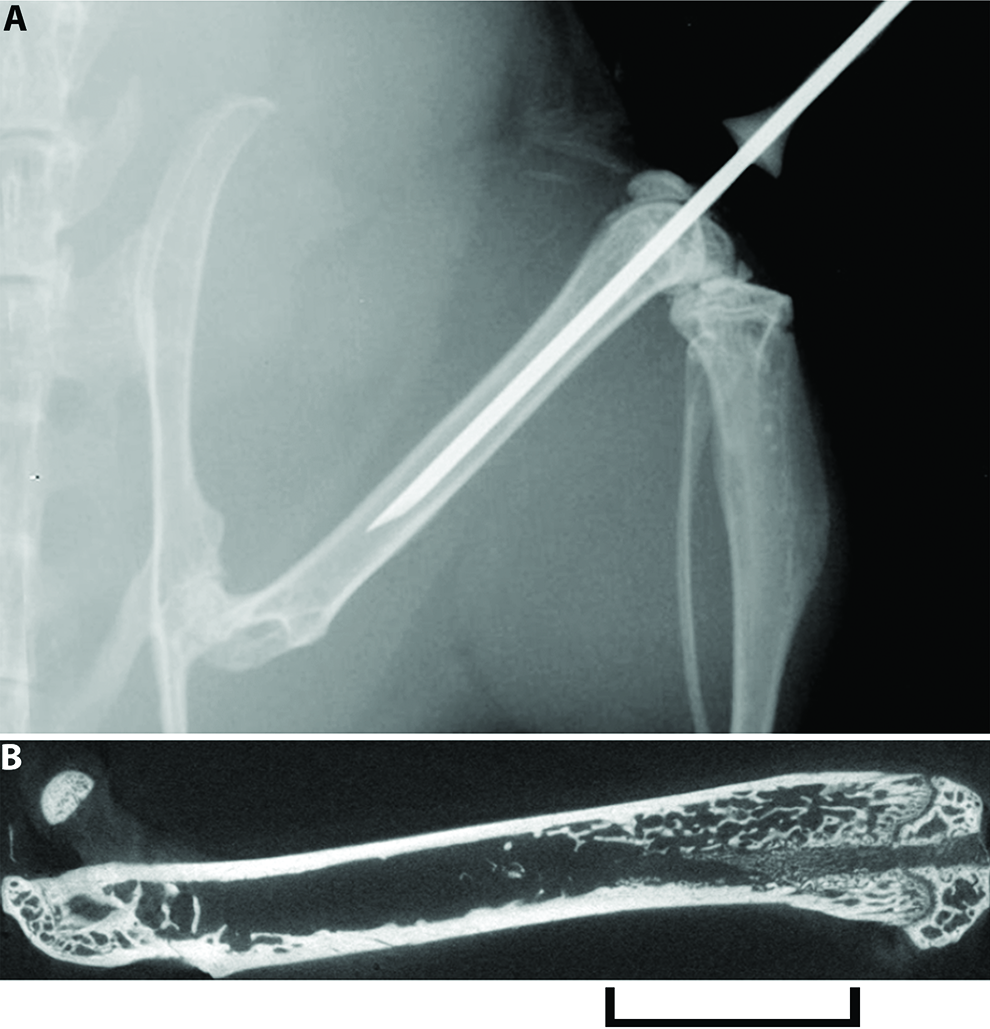

Supplement: Supplementary file 7 — Figure S6 [file 41536_2017_37_MOESM7_ESM.tif]
